# A Prognostic Model Based on NSUN3 Was Established to Evaluate the Prognosis and Response to Immunotherapy in Liver Hepatocellular Carcinoma

**DOI:** 10.1155/2023/6645476

**Published:** 2023-04-18

**Authors:** Jianlin Zhu, Junxi Kuang, Yi Yang, Lei Zhang, Bo Leng, Risheng She, Ling Zou

**Affiliations:** ^1^Dongguan Institute of Clinical Cancer Research, The Tenth Affiliated Hospital of Southern Medical University, China; ^2^Dongguan Key Laboratory of Precision Diagnosis and Treatment for Tumors, The Tenth Affiliated Hospital of Southern Medical University, China; ^3^Department of Cardiovascular Medicine, The Third Affiliated Hospital of Sun Yat-sen University, China; ^4^Department of Emergency, The Tenth Affiliated Hospital of Southern Medical University, China

## Abstract

It is difficult for traditional therapies to further improve the prognosis of hepatocellular carcinoma (LIHC), and immunotherapy is considered to be a promising approach to overcome this dilemma. However, only a minority of patients benefit from immunotherapy, which greatly limits its application. Therefore, it is particularly urgent to elucidate the specific regulatory mechanism of tumor immunity so as to provide a new direction for immunotherapy. NOP2/Sun RNA methyltransferase 3 (NSUN3) is a protein with RNA binding and methyltransferase activity, which has been shown to be involved in the occurrence and development of a variety of tumors. At present, the relationship between NSUN3 and immune implication in LIHC has not been reported. In this study, we first revealed that NSUN3 expression is upregulated in LIHC and that patients with high NSUN3 expression have a poor prognosis through multiple databases. Pathway enrichment analysis demonstrated that NSUN3 may be participated in cell adhesion and cell matrix remodeling. Next, we obtained a set of genes coexpressed with NSUN3 (NCGs). Further LASSO regression was performed based on NCGs, and a risk score model was constructed, which proved to have good predictive power. In addition, Cox regression analysis revealed that the risk score of NCGs model was an independent risk factor for LIHC patients. Moreover, we established a nomogram based on the NCGs-related model, which was verified to have a good predictive ability for the prognosis of LIHC. Furthermore, we investigated the relationship between NCGs-related model and immune implication. The results implied that our model was closely related to immune score, immune cell infiltration, immunotherapy response, and multiple immune checkpoints. Finally, the pathway enrichment analysis of NCGs-related model showed that the model may be involved in the regulation of various immune pathways. In conclusion, our study revealed a novel role of NSUN3 in LIHC. The NSUN3-based prognostic model may be a promising biomarker for inspecting the prognosis and immunotherapy response of LIHC.

## 1. Introduction

Liver hepatocellular carcinoma (LIHC) is a highly lethal malignancy originating from the digestive system and one of the leading causes of cancer-related deaths worldwide [1]. Global cancer epidemiological statistics in 2020 show that there are about one million new cases of liver cancer, most of which are LIHC [2]. Various risk factors are known to predispose to the development of liver cancer, including chronic hepatitis virus infection, aflatoxin B, and alcoholism [3, 4]. Currently, surgical resection is still the preferred treatment option for LIHC. Unfortunately, the overall recurrence rate of LIHC remains high, with a 5-year survival rate of less than 50% [5]. In addition to classic surgical resection, other treatment options for LIHC including ablation, catheterization, and noncatheterization have been widely applied, but the 5-year survival is still less than 20% [6–8]. Traditional treatment strategies are extremely limited to further improve the prognosis of LIHC [9]. Therefore, elucidating the pathogenesis of LIHC is crucial for its treatment.

With the breakthrough of immunotherapy theory of tumor in recently years, immune checkpoint inhibitors (ICIs) based on various immune checkpoints have been developed, and they have brought new hope to LIHC patients [10]. At present, a variety of ICIs have been used in the treatment of LIHC, including PD1 antibody and CTLA-4 antibody [3]. However, in the practical application of ICIs, only a minority of patients benefit from ICIs treatment [11]. Several studies have shown that the level of leukocyte infiltration in the tumor immune microenvironment (TIM) is closely related to the efficacy of immunotherapy [12, 13]. Therefore, it is crucial to explore the specific situation and mechanism of TIM in LIHC for the guidance of LIHC immunotherapy.

NOP2/Sun RNA methyltransferase 3 (NSUN3) is a protein-coding gene with RNA binding and methyltransferase activities [14]. It has been reported that NSUN3-mediated m5C modification of mitochondrial tRNA enhances energy supply by promoting protein synthesis in the mitochondrial respiratory chain, thereby promoting cancer cell invasion and metastasis [14]. Currently, there is no report on the relationship between NSUN3 and immune function in LIHC.

In this study, we first found that NSUN3 was highly expressed in LIHC and correlated with poor patient prognosis. We constructed a prognostic model based on NSUN3 coexpressed genes (NCGs) and validated its applicability for evaluating the prognosis and immunotherapy response of LIHC. Our study revealed a novel role of NSUN3 in LIHC, which may be a potential therapeutic target.

## 2. Materials and Methods

### 2.1. Acquisition of LIHC Transcriptional Data

LIHC transcription data downloaded from The Cancer Genome Atlas (TCGA, https://portal.gdc.cancer.gov/) and GEO (https://www.ncbi.nlm.nih.gov/geo/) public databases. We merged the obtained survival information of LIHC samples with transcriptome data and finally got 342 LIHC samples with survival information (TCGA).

### 2.2. Construction of Risk Scoring Model

We performed LASSO regression analysis on the coexpressed genes of NSUN3. Risk score was assigned to each LIHC patient according to the following established formula: risk score = (*βi*^∗^ Expi), where Expi refers to the expression level of the target gene and *βi* represents the coefficient of the target gene. The ROC curve was implemented to evaluate the predictive ability of the model.

### 2.3. Analysis of Immune Cell Infiltration in LIHC

In this study, we applied CIBERSORT to assess the immune cell infiltration ratio. CIBERSORT is a novel algorithm that relies on a gene expression matrix file called LM22, which can analyze immune cells by identifying and counting specific genes in them [15].

### 2.4. Evaluation of the Correlation between Risk Scores and Immune Profiles

In this study, we applied IPS, TIDE, and ESTIMATE to analyze the correlation of risk score and immune profile. The immunophenoscore (IPS) is a method for predicting response to immune checkpoints by quantifying tumor immunogenicity. The method incorporates multiple parameters, such as immunomodulators, effector cells, and suppressor cells, by weighted quantification of these components, resulting in a final IPS score [16]. ESTIMATE (Estimation of Stromal and Immune cells in Malignant Tumor Tissues using Expression Data) is a novel algorithm to infer tumor tissue components from unique characteristic genes in tumor tissue transcriptional data. In this study, we conducted the ESTIMATE algorithm to analyze the correlation of immune and stromal scores with risk scores [17]. Tumor immune dysfunction and rejection (TIDE) is a predictor of patient response to immune checkpoint inhibitors. Patients with low TIDE scores may be more responsive to immunotherapy, whereas patients with high TIDE scores may respond less to immunotherapy [18].

### 2.5. Statistics

In this study, R software (4.2.2) was applied for calculation and statistical analysis. *p* < 0.05 was considered statistically significant.

## 3. Results

### 3.1. NSUN3 Is Highly Expressed and Associated with Poor Prognosis in LIHC

**Figure 1:**
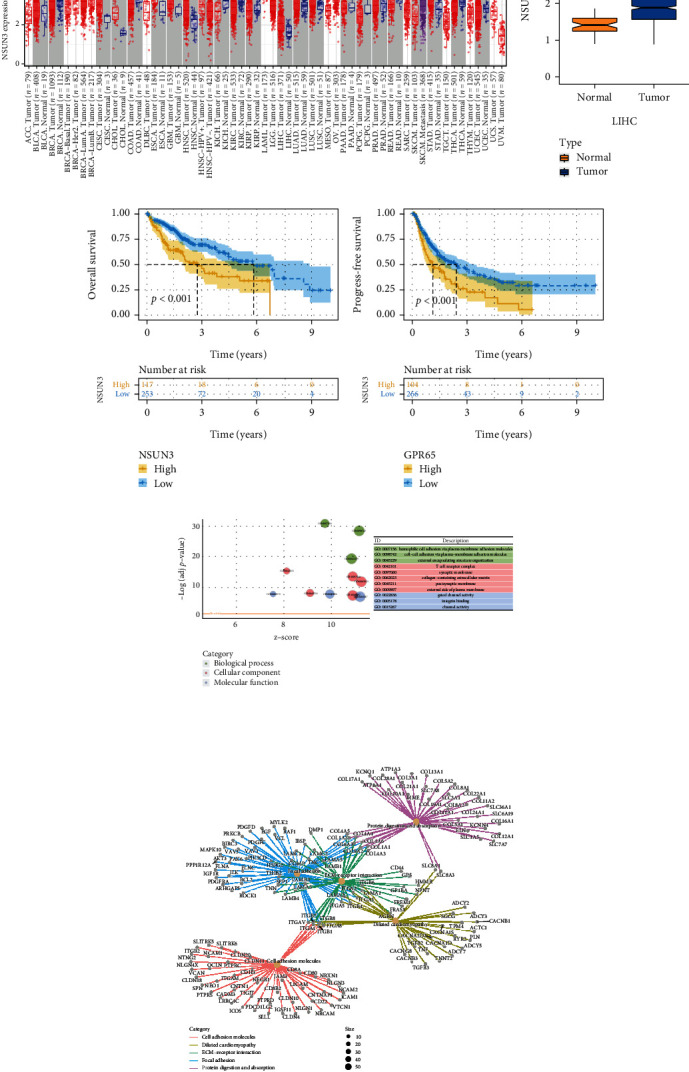
Analysis of NSUN3 expression and prognosis in LIHC and pathway enrichment analysis. (a) Expression level of NSUN3 in pan-cancer (TIMER database). (b) Differential expression of NSUN3 in LIHC and normal tissues. (c, d) Effects of NSUN3 expression level on overall survival (OS) and progression-free survival (PFS). (e) GO analysis based on NSUN3 expression level. (f) KEGG analysis based on NSUN3 expression level.

We first analyzed the mRNA expression levels of NSUN3 in pan-cancer via the TIMER database, and the result showed that NSUN3 was significantly upregulated in LIHC (Figure 1(a)). Next, we further explored the expression of NSUN3 in TCGA-LIHC and found that it was significantly elevated in tumor tissues (Figure 1(b)). In addition, we performed survival analysis based on NSUN3 expression, and the results demonstrated that NSUN3 was closely associated with poor prognosis (Figures 1(c) and 1(d)). Moreover, we applied GO and KEGG enrichment analyses to preliminarily explore the potential role and mechanism of NSUN3 in LIHC. GO analysis showed that the function of NSUN3 was mainly enriched in extracellular matrix remodeling (Figure 1(e)), and KEGG pathway analysis revealed that NSUN3 was enriched in cell adhesion, extracellular matrix remodeling, and focal adhesion junctions (Figure 1(f)). These pathways obtained above suggested that NSUN3 plays an important role in the regulation of LIHC immune function.

### 3.2. Coexpression Gene Analysis of NSUN3

**Figure 2:**
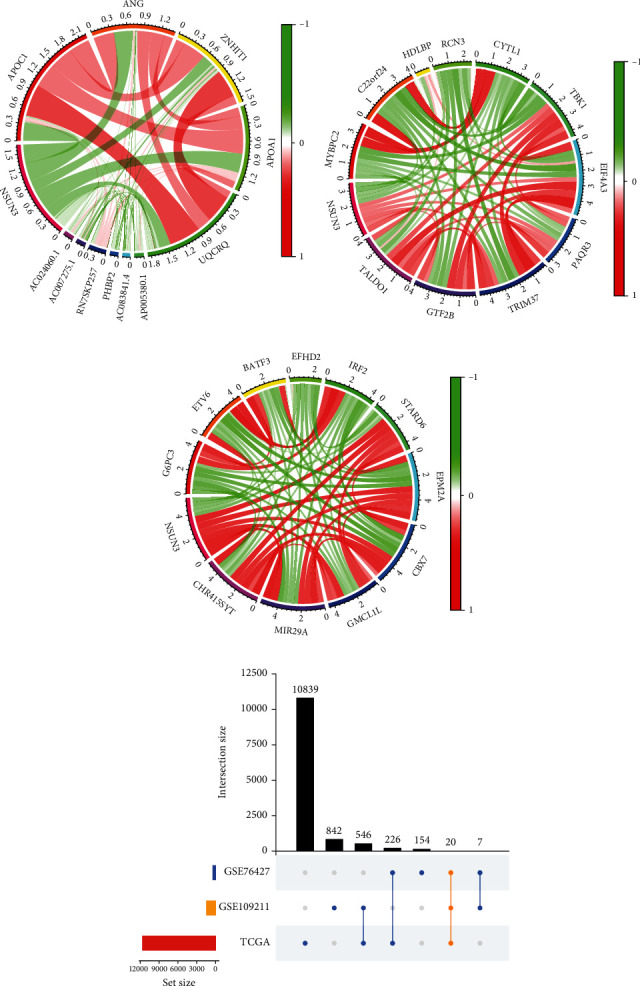
Analysis of NSUN3 coexpressed genes (NCGs). (a–c) Coexpressed genes of NSUN3 in different transcriptome datasets (GSE76427, GSE109211, and TCGA-LIHC cohorts). (d) Intersection of NSUN3 coexpressed genes obtained from different datasets.

The previous data strongly suggested a strong correlation between NSUN3 levels and prognosis, we intended to investigate the coexpressed genes of NSUN3 in LIHC. We analyzed the coexpressed gene network of NSUN3 by 3 LIHC datasets (GSE76427, GSE109211, and TCGA), and the correlation coefficient was set at 0.2 (*p* < 0.05). We presented the top 10 most correlated genes in these three datasets in Figures 2(a)–2(c). Next, the upset plot was applied to intersect the coexpressed genes of the three datasets, and 20 coexpressed genes were obtained finally (Figure 2(d)).

### 3.3. Construction of a Model Based on NSUN3 Expression Levels in LIHC

**Figure 3:**
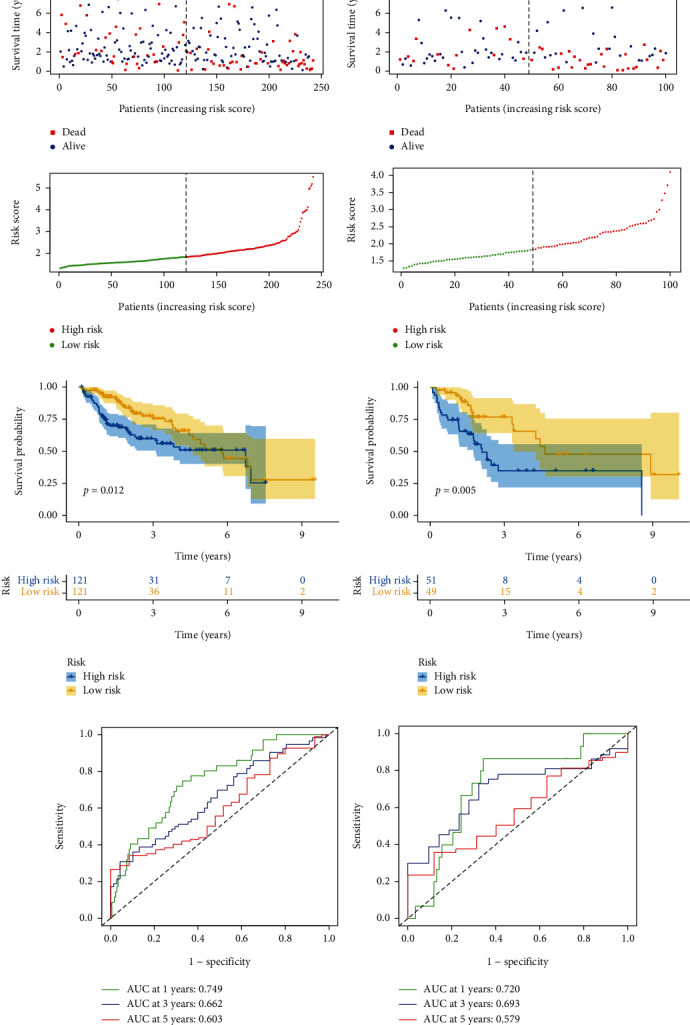
Construction and validation of a prognostic model based on NCGs. (a) Distribution of survival status (upper) and risk scores (lower) for the training datasets. (b) Distribution of survival status (upper) and risk scores (lower) for the validation datasets. (c, d) The Kaplan-Meier curves of overall survival for the high- and low-risk groups in the training and validation datasets. (e, f) Time-dependent receiver operating characteristic curves for the risk score in the training and validation datasets.

We previously obtained a gene set with 20 genes coexpressed with NSUN3. Next, we performed LASSO regression analysis to screen this gene set and obtained 6 coexpression genes with associated NSUN3 (NCGs). Furthermore, based on the obtained 6 genes, we constructed a risk prognostic model based on NSUN3 coexpressed genes in LIHC and randomly divided the LIHC cohort in TCGA into two cohorts at a ratio of 7 : 3, namely, training cohort and validation cohort queues. As shown in Figures 3(a) and 3(b), we risk scored and ranked these patients and found consistent changes in mortality and risk scores among patients. In addition, we further verified that there were significant prognostic differences between high- and low-risk patients in this model (Figures 3(c) and 3(d)). Finally, we evaluated the predictive power of the training and validation cohorts by ROC curves. The AUC of the training cohort at years 1, 3, and 5 was 0.749, 0.662, and 0.603, respectively; the AUC of the validation cohort at years 1, 3, and 5 was 0.720, 0.693, and 0.579, respectively (Figures 3(e) and 3(f)). These data strongly indicated that the model has good predictive performance.

### 3.4. Construction and Verification of Nomogram Based on Predictive Model

**Figure 4:**
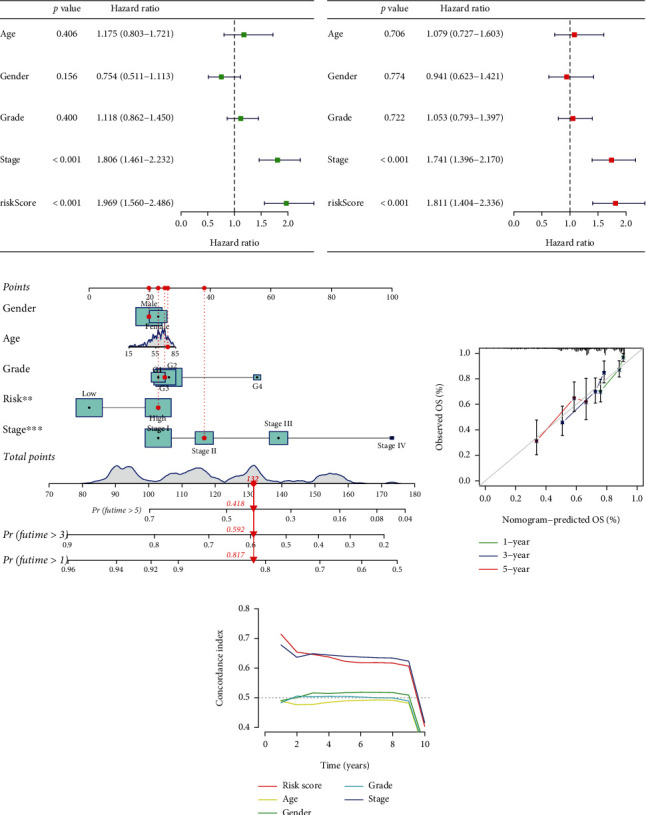
Construction and validation of nomogram based on NCGs-related model and clinical features. (a, b) Univariate and multivariate regression analyses of NCGs model risk score and clinical characteristics. (c) Construction of risk score and nomogram of various clinical characteristics based on NCGs-related model. (d) Validation of the predictive power of the nomogram at years 1, 3, and 5. (e) Analyze the predictive power of the model's risk score by the C-index.

We first performed univariate and multivariate analyses on the risk model, and the results both showed that the HR values of the risk score of the model were 1.969 and 1.871, respectively (Figures 4(a) and 4(b)). Next, we constructed a nomogram integrating the prognostic model and its multiple clinical features, including gender, age, histological grade, and pathological stage (Figure 4(c)). Meanwhile, we verified the accuracy of the nomogram, and the results showed that the nomogram had accurate predictive capacity (Figure 4(d)). Interestingly, the accuracy of the risk model that we further evaluated by the C index also demonstrated good performance in the assessment of LIHC prognosis (Figure 4(e)).

### 3.5. Correlation between NCG-Related Model Risk Scores and Immune Microenvironment in LIHC

**Figure 5:**
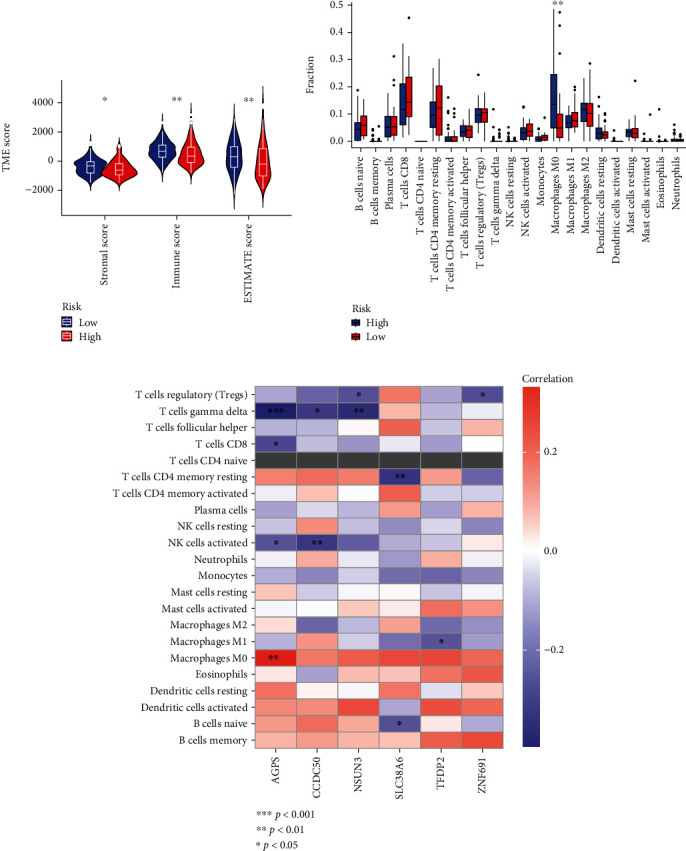
Analysis of the TIM of LIHC via risk scores of NCGs-related model. (a) Correlation between high and low NCGs risk score and TME score. (b) Correlation between high and low NCGs risk scores and 21 types of immune cell infiltration. (c) Correlation between NSUN3 coexpressed genes and immune cell infiltration.

Immune cells in the tumor immune microenvironment (TIM) induce immune escape by interacting with tumor cells [13]. To clarify their complex relationship, we applied the ESTIMATE algorithm to analyze the TIM of LIHC and observed the differences in matrix score, immune score, and comprehensive score between the high-risk group and the low-risk group, respectively. The results demonstrated that the TME score of the low-risk group was significantly higher than in the high-risk cohort (Figure 5(a)). Next, we investigated the infiltration abundance of 21 immune cells in high- and low-risk patients by the CIBORESORT algorithm, and the results showed that the infiltration abundance of M0 macrophage cells in the low-risk group was higher than that in the high-risk group (Figure 5(b)). In addition, we further explored the correlation between 6 NCGs genes and 21 types of immune cells, the results revealed that AGPS was positively correlated with M0 macrophages but negatively correlated with gamma delta T cells and CD8 T cells; CCDC50 was negatively correlated with activated NK cells and *γδ*T cells; NSUN3 was negatively correlated with Treg cells and *γδ*T cells; SLC38A6 was negatively correlated with naive B cells and memory resident CD4^+^ T cells; TFDP2 was negatively correlated with M1 macrophages; ZNF691 was negatively correlated with Treg cells (Figure 5(c)).

### 3.6. Risk Scores for NCGs-Related Models Predict Immunotherapy Response

**Figure 6:**
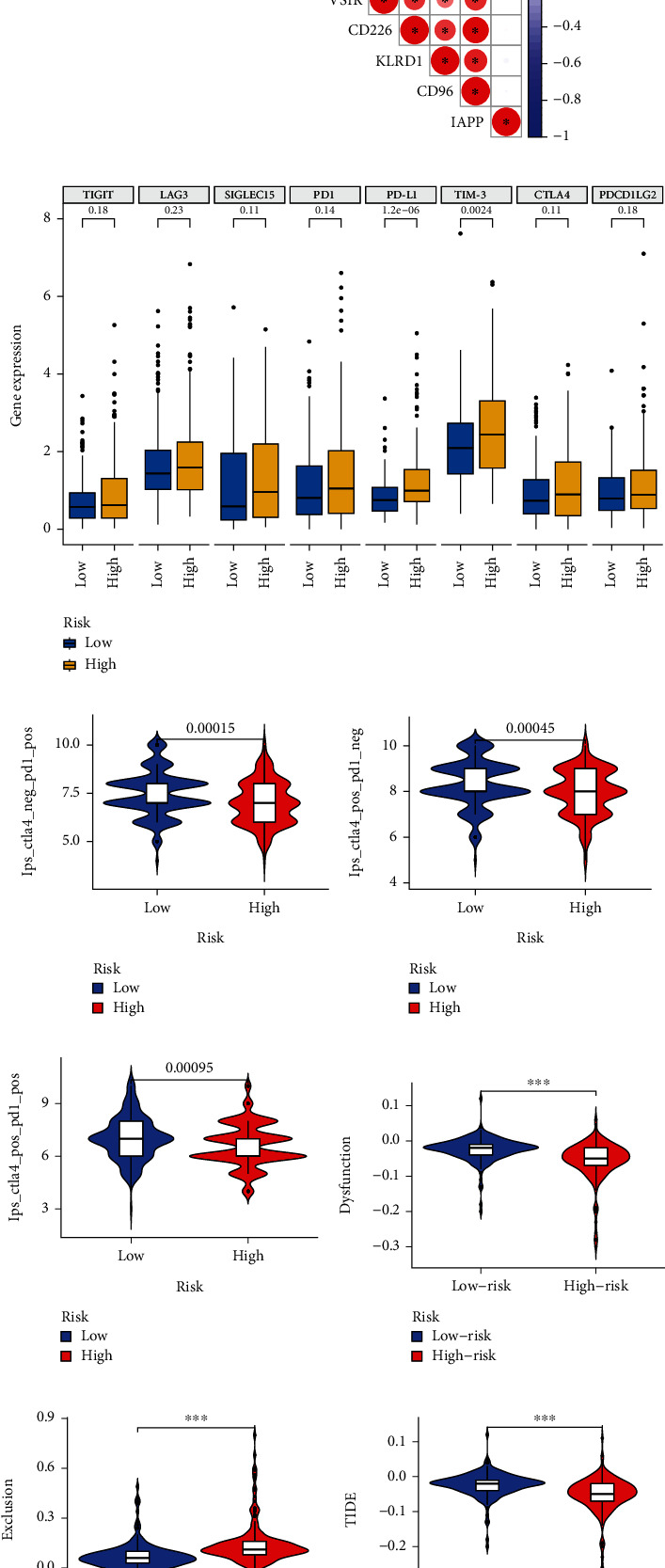
Risk scores from NCGs-related model predict LIHC response to immunotherapy. (a) Correlation of NCGs risk score with multiple immune checkpoints. (b) Differences in expression of multiple immune checkpoints in the high- and low-risk groups. (c) In the case of CTLA4 negative but PD1 positive, the high-risk group had lower IPS. (d) In the case of CTLA4 positive but PD1 negative, the high-risk group had lower IPS. (e) In the case of CTLA4 and PD1 negative, the high-risk group had lower IPS. (f) Low-risk patients have more pronounced immune dysfunction. (g) High-risk patients have a stronger tendency to immune exclusion. (h) Low-risk patients have lower TIDE scores.

Immune checkpoints play a key role in the regulation of immune cell function and are important predictors for assessing immunotherapy response. Therefore, in the present study, we first analyzed the correlation between 11 immune checkpoints and risk scores of NCGs-related models, and the results showed that the risk scores of the NCGs-related models were strongly associated with most immune checkpoints (Figure 6(a)), except for KLRD1 and IAPP. As shown in Figure 6(b), we further explored the differences of 8 immune checkpoints in high- and low-risk cohorts, and the results revealed that PD-L1 and TIM-3 were significantly higher in high-risk cohorts than in low-risk cohorts. Furthermore, given the strong correlation between the risk score of the NCG-related model and immune checkpoints, we explored whether the risk score of this model could predict the response of LIHC patients to treatment with ICIs. In addition, IPS and TIDE have been widely used to assess tumor response to immunotherapy in recent years. The results of our analysis demonstrated that in the low-risk score group, PD1-positive and CTLA4-negative patients had significantly higher IPS; interestingly, high-risk patients had significantly lower IPS scores in CTLA-positive and PD1-negative patients (Figures 6(c)–6(e)). Finally, the results of the TIDE algorithm implied that the low-risk group had higher immune dysfunction than the high-risk group, while the immune exclusion was lower than that of the high-risk group (Figures 6(f) and 6(g)).

### 3.7. Enrichment Analysis of NCGs-Related Model

**Figure 7:**
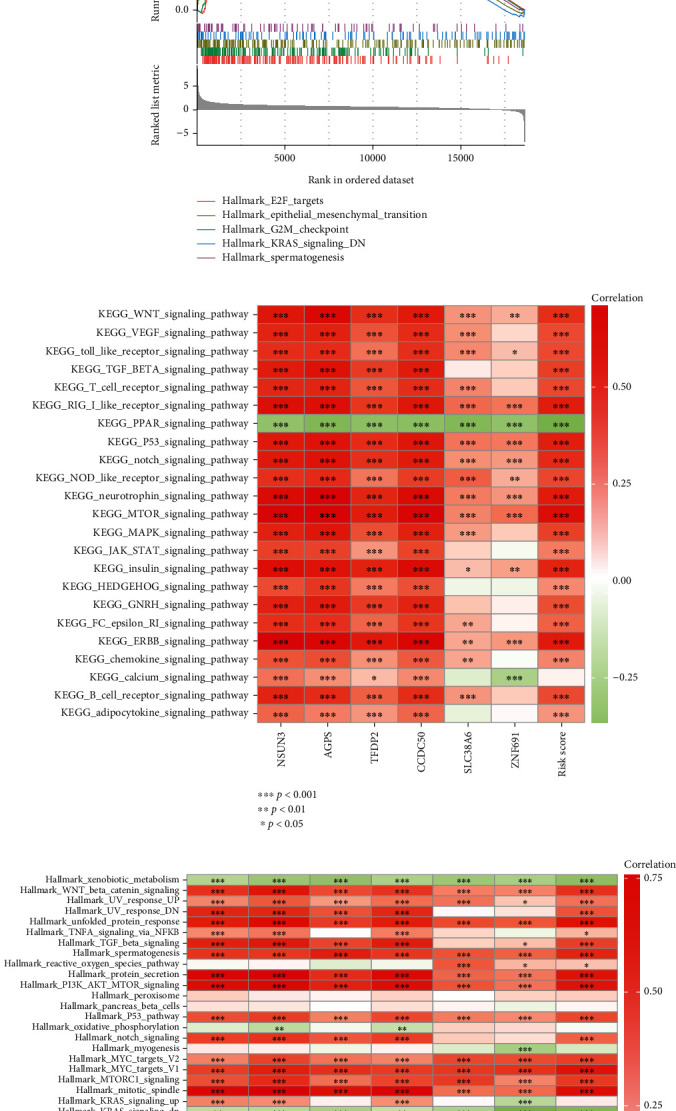
Enrichment analysis of NCG-related model. (a) KEGG pathway enrichment analysis based on the risk score of NCG-related model. (b) HALLMARKER enrichment analysis based on the risk score of NCG-related model. (c) Correlation analysis between NCGs and KEGG enrichment pathway. (d) Correlation analysis between NCGs and Hallmark enrichment pathway.

Our previous studies revealed that risk stratification of NCGs-related models in LIHC is closely related to cell infiltration. To explore the underlying mechanism, we performed pathway enrichment analysis on high- and low-risk cohorts of the NCGs-related risk scoring model by the KEGG and HALLMARK gene sets. The results of gene set enrichment analysis showed that cell cycle-related pathways were significantly enriched NCGs-related models (Figures 7(a) and 7(b)). Next, we further analyzed the correlation of each NCGs with the enrichment pathway. The results revealed that in the KEGG gene set, multiple signaling pathways were positively correlated with NCGs, such as WNT, VEGF, TGF-*β*, and NOTCH signaling pathways. In the HALLMARK gene set, multiple signaling pathways were positively correlated with NCGs, such as unfolded protein pathway, KRAS, and angiogenesis (Figures 7(c) and 7(d)).

## 4. Discussion

The occurrence and development of tumors is a multistep process that is regulated by gene network [19]. Immune response is a special situation of inflammatory reactions [20]. The maintenance of normal immune function can effectively eliminate tumors. However, tumors in progress are often accompanied by immune evasion, which in turn induces the distant metastasis of the tumor [21]. Therefore, it is critical to clarify the specific mechanism of tumor immune function to the treatment of malignant tumors. In this study, we first discovered that NSUN3 upregulation was related to poor prognosis in LIHC. Then, by multiple database LIHC cohorts, we constructed a prognostic model based on NSUN3 coexpression genes and confirmed its accuracy and effectiveness. More importantly, we further explored the value of the model. We found that the risk score of NSUN3-related model is related to immune profile and can instruct the choice of immunotherapy.

The prognostic model based on various functional genes has become a hot spot in guiding the prognosis of tumor. Ruan et al. analyzed the expression of ZEB1-AS1 in colorectal cancer and found that its high expression was positively associated with poor prognosis. Furthermore, a prognostic model based on ZEB1-AS1 coexpression gene was constructed. The ROC curve areas of the model in the training cohort were 0.650, 0.706, and 0.706, respectively, and in the validation cohort were 0.705, 0.592, and 0.753, respectively [22]. Li et al. revealed that AHCYL1 acts as an oncogene in colorectal cancer. A prognostic model based on AhCYL1-related genes was constructed. To further explore the effectiveness of the model, the results showed that the areas of the ROC curves of the 1, 3, and 5 years in the training cohort were 0.665, 0.634, and 0.695, and the areas of the ROC curves of the 1, 3, and 5 years in the validation cohort were 0.691, 0.754, and 0.726, respectively [23]. In the present study, we constructed the prognostic model of NUSN3-related genes, and the areas under the ROC curve of 1, 3, and 5 years in the training cohort were 0.749, 0.662, and 0.603, while the areas under the ROC curve of 1, 3, and 5 years in the validation cohort were 0.720, 0.693, and 0.597. This result indicated that the predictive power of our constructed model is not weaker than that of other previous studies.

The prediction of immunotherapy therapy has always been difficult for immunotherapy [24]. The development of models that accurately predict the response to immunotherapy has been a goal we have pursued. In this study, we constructed a prognostic model based on NSUN3-related genes. The level of risk scores of this model shows different responses to immunotherapy. The results further revealed that NSUN3 participated in regulating LIHC immune profile. The data of this study mainly derived from the public database and lacked corresponding clinical evidence support, which requires us to be validated in our follow-up studies.

In conclusion, this study demonstrated a novel role for NSUN3 in regulating the immune implication of LIHC. The development of targeted NSUN3 drugs may be a promising research direction for the treatment of LIHC.

## Data Availability

The data and results in this study are available from the corresponding authors upon reasonable request.
